# Long-Term Survival after Adrenalectomy for Asynchronous Metastasis of Bladder Cancer to the Bilateral Adrenal Glands

**DOI:** 10.1155/2012/425230

**Published:** 2012-11-20

**Authors:** S. Washino, M. Hirai, A. Matsuzaki, Y. Kobayashi

**Affiliations:** Department of Urology, Saitama Medical Center, Jichi Medical University, 1-847 Amanuma Town, Omiya Ward, Saitama Perfecture, Saitama 330-8503, Japan

## Abstract

Isolated adrenal metastasis of bladder cancer, particularly the bilateral, is quite rare. Systemic chemotherapy is the treatment of choice for metastatic urothelial carcinoma. However, despite initially promising response rates of approximately 45%–71%, most tumors eventually show progression, and the median survival time following chemotherapy regimen is approximately 14-15 months. Recently, favorable results of surgery for metastatic urothelial carcinoma have been reported. Here, we report a rare case of asynchronous metastasis of bladder cancer to the bilateral adrenal glands with long-term survival after bilateral adrenalectomy. A 69-year-old man underwent radical cystoprostatectomy and ileal conduit urinary diversion for invasive bladder cancer. Ten months later, left adrenalectomy was performed for a left adrenal tumor, revealing metastatic urothelial carcinoma. After adjuvant chemotherapy, a tumor in the right adrenal gland was detected. Right adrenalectomy was done, and the tumor was also found to be metastatic urothelial carcinoma. The patient had an uneventful recovery after starting steroid replacement therapy. Three years later, he was doing well and had no evidence of recurrence. Adrenalectomy for isolated adrenal metastasis of urothelial carcinoma may be a reasonable option, even if such metastases are bilateral.

## 1. Introduction

The common sites for metastasis of bladder cancer are the pelvic lymph nodes, liver, lung, bone, and adrenal glands. Adrenal metastasis occurs in 14% of patients [1], but isolated adrenal metastasis, particularly the bilateral, is rare.

Here, we report a rare case of asynchronous metastasis of bladder cancer to the bilateral adrenal glands with long-term survival after bilateral adrenalectomy.

## 2. Case Presentation

**Figure 1:**
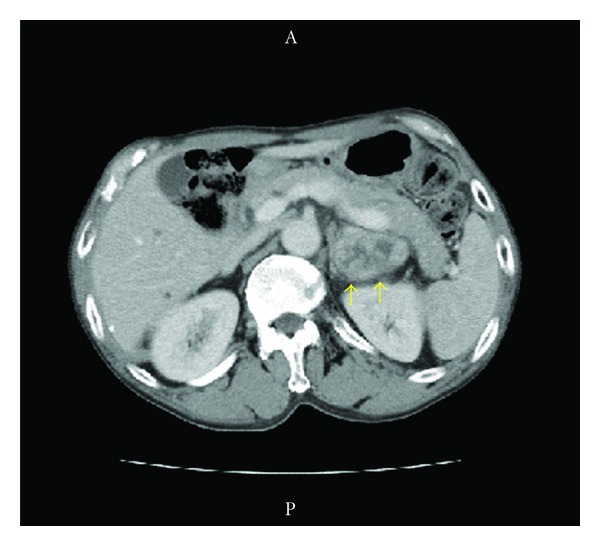
Computed tomography at 8 months after cystectomy shows a left adrenal tumor (5.0 × 4.0 cm).

**Figure 2:**
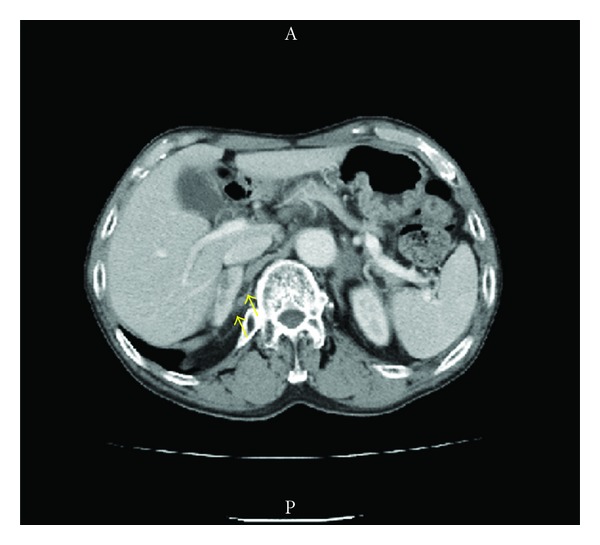
Computed tomography at 6 months after the left adrenalectomy shows right adrenal tumor (5.0 × 1.8 cm).

In July 2007, a 69-year-old man presented with the complaint of voiding dysfunction. Computed tomography (CT) showed a massive tumor in the bladder without lymph node involvement or other metastases. In October 2007, he underwent radical cystoprostatectomy and ileal conduit urinary diversion together with pelvic lymph node dissection. Histopathological examination of the resected specimen revealed high-grade urothelial carcinoma invading to the muscle without lymph node involvement (grade 3 > 2, pT2b, n0). At followup 8 months later, CT detected a mass in the left adrenal gland (Figure 1), which increased in size thereafter. In October 2008, he underwent left adrenalectomy, and metastatic urothelial carcinoma was diagnosed. He received 2 cycles of adjuvant GCD therapy (gemcitabine, cisplatin, and docetaxel) postoperatively. After this chemotherapy, however, CT detected a tumor in the right adrenal gland (Figure 2). Although he received 3 cycles of MEC therapy (methotrexate, epirubicin, and cisplatin), the tumor did not decrease in size. Because there were no apparent metastases other than the right adrenal tumor, he underwent right adrenalectomy, and metastatic urothelial carcinoma was confirmed. The patient had an uneventful recovery on steroid replacement therapy. At followup 3 years later, he was well and had no evidence of recurrence.

## 3. Discussion

Metastasis in the adrenal gland is present in 14% of patients with metastatic bladder cancer [1], but isolated adrenal metastasis arising from bladder cancer is rare. In fact, asynchronous isolated adrenal metastases of bladder cancer have never been reported before, to our best knowledge, and this is the first case. 

According to a recent guideline, systemic chemotherapy is the treatment of choice for metastatic urothelial carcinoma. However, despite initially promising response rates of approximately 45%–71% [2–4], most tumors eventually show progression, and the median survival time following chemotherapy regimen is approximately 14-15 months [2–4]. 

Recently, favorable results of surgery for metastatic urothelial carcinoma have been reported. The median overall survival time of patients who received resection of metastases combined with chemotherapy was 31–42 months, with some patients being alive and disease-free for more than 3 years after metastasectomy [5–7]. Thus, there is a subgroup of patients with metastatic urothelial carcinoma in whom cure can be achieved surgically. Kanzaki et al. reported that solitary metastasis was a factor associated with prolonged survival after resection of pulmonary metastasis from transitional cell carcinoma of the urinary tract [8]. 

Muth et al. reported on a series of 30 patients undergoing adrenalectomy for metastasis and stated that survival benefit demonstrated by potentially curative surgery indicates the therapeutic value of adrenalectomy [9]. 

Our patient had asynchronous isolated adrenal metastasis of urothelial carcinoma after cystectomy. Although 2 systemic chemotherapy regimens (GCD and MEC) had no effects on his adrenal metastasis, the patient has remained free of disease for 3 years after the second adrenalectomy. Wyler reported a patient who was free of disease for two years after adrenalectomy for an adrenal metastasis arising from transitional cell carcinoma of the bladder [10], and such an outcome is consistent with our case.

In conclusion, adrenalectomy may be an option for isolated adrenal metastasis of bladder carcinoma, even when such metastases are bilateral.
